# P38 and JNK Mitogen-Activated Protein Kinases Interact With Chikungunya Virus Non-structural Protein-2 and Regulate TNF Induction During Viral Infection in Macrophages

**DOI:** 10.3389/fimmu.2019.00786

**Published:** 2019-04-12

**Authors:** Tapas Kumar Nayak, Prabhudutta Mamidi, Subhransu Sekhar Sahoo, P. Sanjai Kumar, Chandan Mahish, Sanchari Chatterjee, Bharat Bhusan Subudhi, Soma Chattopadhyay, Subhasis Chattopadhyay

**Affiliations:** ^1^School of Biological Sciences, National Institute of Science Education and Research, HBNI, Bhubaneswar, India; ^2^Infectious Disease Biology, Institute of Life Sciences, Bhubaneswar, India; ^3^School of Pharmaceutical Sciences, Siksha O Anusandhan University, Bhubaneswar, India

**Keywords:** Chikungunya, Alphavirus, MAPK, Macrophages, TNF, p38, JNK, c-jun

## Abstract

Chikungunya virus (CHIKV), a mosquito-borne Alphavirus, is endemic in different parts of the globe. The host macrophages are identified as the major cellular reservoirs of CHIKV during infection and this virus triggers robust TNF production in the host macrophages, which might be a key mediator of virus induced inflammation. However, the molecular mechanism underneath TNF induction is not understood yet. Accordingly, the Raw264.7 cells, a mouse macrophage cell line, were infected with CHIKV to address the above-mentioned question. It was observed that CHIKV induces both p38 and JNK phosphorylation in macrophages in a time-dependent manner and p-p38 inhibitor, SB203580 is effective in reducing infection even at lower concentration as compared to the p-JNK inhibitor, SP600125. However, inhibition of p-p38 and p-JNK decreased CHIKV induced TNF production in the host macrophages. Moreover, CHIKV induced macrophage derived TNF was found to facilitate TCR driven T cell activation. Additionally, it was noticed that the expressions of key transcription factors involved mainly in antiviral responses (p-IRF3) and TNF production (p-c-jun) were induced significantly in the CHIKV infected macrophages as compared to the corresponding mock cells. Further, it was demonstrated that CHIKV mediated TNF production in the macrophages is dependent on p38 and JNK MAPK pathways linking p-c-jun transcription factor. Interestingly, it was found that CHIKV nsP2 interacts with both p-p38 and p-JNK MAPKs in the macrophages. This observation was supported by the *in silico* protein-protein docking analysis which illustrates the specific amino acids responsible for the nsP2-MAPKs interactions. A strong polar interaction was predicted between Thr-180 (within the phosphorylation lip) of p38 and Gln-273 of nsP2, whereas, no such polar interaction was predicted for the phosphorylation lip of JNK which indicates the differential roles of p-p38 and p-JNK during CHIKV infection in the host macrophages. In summary, for the first time it has been shown that CHIKV triggers robust TNF production in the host macrophages via both p-p38 and p-JNK/p-c-jun pathways and the interaction of viral protein, nsP2 with these MAPKs during infection. Hence, this information might shed light in rationale-based drug designing strategies toward a possible control measure of CHIKV infection in future.

## Introduction

Chikungunya virus (CHIKV), a mosquito-borne Alphavirus belongs to Togaviridae family, is transmitted through either *Aedes aegypti* or *Aedes albopictus* mosquito. CHIKV mediated disease is one of the global challenges due to its endemics in different parts of the world (103 countries), such as Tanzania (1–3), Reunion island (4–7), India (8–12), Italy (13, 14), and Thailand (15–18). Among Alphaviruses, CHIKV is considered as one of the most successfully evolved virus. The Arboviruses including CHIKV have been evolving and re-emerging from centuries and their emergence and dispersion are more rapid and geographically extensive. This might be due to increase in global communication, mass immigration, vector adaptation to urbanization and land perturbation (19). Even though mortality due to CHIKV is very rare and restricted to children's (below 1 year), old age (above 65 years) or immune compromised patients, the pathogenesis (mainly inflammatory responses) may persist for very long periods of time both in humans and macaque model (20, 21). Currently, arboviruses raise a serious threat to the global public health, due to unavailability of effective drugs or vaccines (22, 23).

Recent studies on CHIKV induced immune responses suggest that the host immune system is found to be both beneficiary in one hand by controlling viral infection, whereas deleterious on the other hand by promoting severe inflammatory responses (24–28). Studies have shown that CHIKV induces different inflammatory cytokines/chemokines (TNF, IL-1β, IL-6, IFN-γ, IL-8, and MCP-1) (24, 29–37), which might be associated with arthritis like pathogenesis during CHIKV infection. In different *in vivo* systems (both mouse and non-human primates), predominant cellular infiltration of macrophages, monocytes, NK cells and T cells to the site of inoculation and other tissues have been observed (38, 39). Moreover, immunohistochemistry and flow cytometry based analysis of muscles and synovial biopsies revealed that macrophages are major infiltrating cells among MPS (mononuclear phagocytic system) (25, 40). Blood monocytes and tissue macrophages are the major immune cells infected by CHIKV (21, 31, 41). In macaque, synovial macrophages have been identified as the major host cell for long-term viral persistence (21). This productive infection of CHIKV in the host macrophages might be associated with arthritis like pathogenesis despite robust immune activation (41, 42).

T cell immune responses specific to CHIKV is not clearly understood yet. Teo TH et al. have suggested that CD4^+^ T cells (but not CD8^+^ T cells) are essential for the development of CHIKV induced pathogenesis without affecting virus infection and dissemination in mice and this is independent of IFN-γ (43). Flow cytometry based analysis of circulating lymphocytes in CHIKV patients confirms that there are both CD4^+^ and CD8^+^ T cell responses during early and late phases of infection, respectively. Moreover, CD95 mediated apoptosis was also detected in CD4^+^ T cells after 2 days of symptom appearance (44), which might be one of the strategies to evade host immunity. Purified T cells (both CD4^+^ and CD8^+^) from the chronic and recovered patients from 2005 to 2006 La Reunion islands showed immune activation when challenged with synthetic CHIKV peptides and inactivated virus particles (42). The DNA vaccine based on the consensus sequences of E1/2 and capsid protein (with several modifications) of CHIKV resulted in robust IFN-γ and IgG production suggesting that CHIKV induces both T and B cell specific responses (45, 46).

There are three major studied ser/thr kinases under the mitogen-activated protein kinase (MAPK) family, such as p38, JNK, and ERK, which are known to regulate multiple cellular pathways such as cell proliferation, activation, inflammation, cytokine and chemokine productions and different pathological conditions (47–53). In addition, activation of MAPKs by different pathogens and other inflammatory diseases have been reported to induce pro-inflammatory cytokines such as TNF in the host cells (48–51, 53, 54). The MAPKs have been shown to be activated by phosphorylation in specific positions (Ser/Tyr/Thr) by several viral infections, such as coronavirus type 2, Hepatitis C virus, Rhinovirus and Epstein-Barr virus (54–58). CHIKV is also known to induce MAPKs during infection in various non-immune cells and treatment of an alkaloid berberine, reduces viral infection and joint swelling in mice (59, 60).

We have shown earlier that CHIKV triggers robust TNF production in the host macrophages, which might be a key mediator of virus induced inflammation (37) and macrophages are identified as the major cellular reservoirs during the late stages of CHIKV infection in macaques (21). However, the precise role of MAPK activation pathways in terms of CHIKV infection and associated robust TNF induction in macrophages (immune cell) remains largely unknown. Hence, an attempt was made to understand the involvement of MAPKs in CHIKV infection and TNF induction in the host macrophages.

## Materials and Methods

### Cells and Viruses

DRDE-06 (accession no. EF210157.2), an Indian outbreak strain of CHIKV and Vero cells (African green monkey kidney epithelial cell line) were kind gifts from Dr. M. M. Parida, DRDE, Gwalior, India. The mouse monocyte/macrophage cell line, Raw264.7 (ATCC® TIB-71™) was maintained in RPMI-1640 (HiGlutaXL™ RPMI-1640) supplemented with 2.0 mM L-glutamine, Penicillin 100 U/ml, Streptomycin 0.1 mg/ml (Himedia Laboratories Pvt. Ltd, MH, India), 10% Fetal bovine serum (FBS; PAN Biotech, Germany) at 37°C under a humidified incubator with 5% CO_2_. The Vero cells were maintained in Dulbecco's modified Eagle's medium (DMEM; PAN Biotech, Germany) supplemented with 5% FBS, Gentamycin (Sigma-Aldrich, MO, USA). The enzyme-free cell dissociation reagent (ZymeFree™; Himedia Laboratories Pvt. Ltd, MH, India) was used for the maintenance of the Raw264.7 cells.

Eight to ten weeks old male or female BALB/c mice were used for this experiment. The animals used in these experiments were approved by Institutional Animal Ethics Committee, NISER and followed the guidelines by Committee for the Purpose of Control and Supervision of Experiments on Animals (CPCSEA).

### Antibodies and Reagents

The mouse anti-CHIKV-nsP2 antibody used in the current study was developed by us (61). Anti-mouse CD3 antibody, anti-TNF antibody, anti-CD69 FITC, HRP linked anti-mouse, and HRP linked anti-rabbit secondary antibodies were purchased from BD Biosciences (CA, USA). Anti-mouse CD28 and CD90.2 APC were procured from Tonbo Biosciences (CA, USA). The monoclonal antibodies for p38, p-p38, JNK, p-JNK, ERK1/2, p-ERK1/2, p-IRF3, and p-c-jun were purchased from cell signaling technology (MA, USA). The anti-mouse Alexa Fluor 488 and anti-rabbit Alexa Fluor 647 were purchased from Invitrogen (CA, USA). The rabbit polyclonal antibodies against p-p38 and p-JNK used for immunoprecipitation were purchased from Santa Cruz biotechnology (TX, USA). Mouse IgG, rabbit IgG isotype control, and anti-GAPDH antibody were purchased from Abgenex India Pvt. Ltd (OD, India). Saponin and Bovine serum albumin fraction V were purchased from Sigma-Aldrich (MO, USA). SB203580 (p-p38 inhibitor, SB), and SP600125 (p-JNK inhibitor, SP) were purchased from Merck Millipore (MA, USA).

### MTT Assay

MTT assay was performed to assess the cytotoxicity of SB and SP according to the methods described before (37). Briefly, the Raw264.7 cells were seeded in 96 well plates at a density of 5 × 10^3^ cells per well before 18–20 h of drug treatment. Then, the cells were washed in 1X PBS and incubated with different concentrations of drugs in triplicate. As both SB and SP were dissolved in the Dimethyl Sulfoxide (DMSO), it was taken as solvent control. After 12 h, the cells were incubated with the MTT reagent to a final concentration of 10% (v/v) in RPMI media. Then, the cells were placed in the incubator for upto 2 h for the formation of visible crystals. Later, the media (containing MTT) were removed without disturbing the cells and 100 μl of solubilization solution was added per well followed by incubation for 15 min at room temperature (RT). The percent viable cells were calculated after taking the absorbance of the solution at 550 nm by Microplate Reader (Bio-Rad, CA, USA).

### CHIKV Infection in Macrophage

Raw 264.7 cell line has been well-reported to study CHIKV infection, replication and associated altered host immune responses (31, 37). The Raw264.7 cells were seeded in six-well cell culture plates before 18–20 h of infection with around 70% confluency. The cells were infected with the DRDE-06 strain of CHIKV with multiplicity of infection (MOI) 5 as reported previously (37). Briefly, after washing the cells in 1X PBS, the virus was added over confluent monolayer for 2 h in the incubator with manual shaking at an interval of 15 min. Then, the virus inoculum was washed in 1X PBS to remove unbound viruses and the cells were maintained in the complete RPMI-1640 media. The infected cells and the supernatants were collected at different time points and subjected to further processing according to the assay.

SB and SP treatments were given as described before (62). Briefly, cells were pretreated with the desired concentrations of SB, SP or DMSO for 2 h in serum free media (SFM). Then the infection was carried out in the presence of either solvent control (DMSO), SB or SP. The cells were washed thoroughly with 1X PBS after 2 h and cultured in SFM containing the drug for 3 h. Then, serum was added to the cells and maintained in the incubator until harvesting (37).

### Plaque Assay

Viral plaque assay was performed to determine the titer of CHIKV as described previously (10). In brief, after infecting the Vero cells with different dilutions of cell culture supernatants (collected from CHIKV infected Raw cells), the cells were overlaid with complete DMEM containing methyl cellulose and maintained in the incubator. After the development of the visible plaques (usually 4–5 days), the cells were fixed in formaldehyde at room temperature, washed gently in tap water and stained with crystal violet. Then, the numbers of plaques were counted manually under white light.

### Flow Cytometry (FC)

Flow cytometric assay was carried out as reported previously (37). Briefly, both mock and CHIKV infected Raw264.7 cells were harvested and fixed in 4% paraformaldehyde for 10 min at RT. Then, the cells were re-suspended in FACS buffer and stored at 4°C until staining. For intracellular staining (ICS), the cells were permeabilized in freshly prepared 1X permeabilization buffer followed by blocking buffer (1% BSA in permeabilization buffer) for 30 min at RT. Then, the cells were incubated with different primary antibodies for 30 min at RT, followed by washing with 1X permeabilization buffer twice. After that, the cells were incubated in Alexa Fluor® 488 and Alexa Fluor® 647 conjugated secondary antibodies followed by washing with 1X permeabilization buffer. The mouse IgG and rabbit IgG were taken as isotype control during ICS. The FcR blocking reagent (Miltenyi Biotec, Bergisch Gladbach, Germany) was used prior to the primary antibody incubation to prevent non-specific binding of antibodies to the Fc receptors on macrophages. Then, the cells were acquired by the BD FACS Calibur^TM^ flow cytometer (BD Biosciences, CA, USA) and analyzed by the CellQuest Pro software (BD Biosciences, CA, USA). A total of approximately 10 × 10^3^ cells were acquired per sample.

### Sandwich ELISA for Cytokine Analysis

TNF production from the macrophage cell culture supernatants was quantified by the BD OptEIA™ sandwich ELISA kit (BD Biosciences, CA, USA) according to the manufacturer's instructions (37). The cytokine concentrations in the test samples were calculated in comparison with the corresponding standard curve prepared by using different concentrations of the recombinant TNF in pg/ml.

### Western Blot Analysis

Western blot analysis was performed to assess the levels of different protein expressions according to the protocol mentioned before (37). In brief, both the mock and CHIKV infected cells were washed with ice-cold 1X PBS and the whole cell lysate (WCL) was prepared by Radio Immuno Precipitation Assay (RIPA) lysis buffer. The protein concentration was quantified by the Bradford reagent (Sigma-Aldrich, MO, USA). Equal amount of protein was loaded in the 10% SDS-PAGE after mixing with 2X Laemmli buffer (1:1) and blotted on to a PVDF membrane (Millipore, MA, USA). Then the transferred membranes were blocked with 3% BSA followed by overnight incubation with different primary antibodies. Then, the membranes were thoroughly washed with TBST and incubated with the HRP conjugated secondary antibodies for 2 h at RT. After washing with TBST, the blots were subjected to chemiluminescence detection by the Bio-Rad gel doc with the Quantity One software (Bio-Rad, CA, USA). For band intensity quantification, Western blot images were subjected to further analysis by the Quantity One 1-D analysis software while normalizing to the corresponding GAPDH loading control.

### Co-immunoprecipitation

Raw cells were infected with CHIKV as described above and harvested at 6 hpi. The cells were lysed with NP-40 (Nonidet P-40) lysis buffer (250 mM NaCl, 5 mM EDTA, 10% glycerol, 1% NP-40, 50 mM Tris, pH 7.4, supplemented with protease inhibitor and phosphatase inhibitor cocktail). The resultant whole cell lysates were subjected to immunoprecipitation by Immunoprecipitation Kit Dynabeads® Protein A (Thermo Fisher Scientific, MA, USA) according to the manufacturer's instructions. Briefly, both the mock and CHIKV infected whole cell lysates were incubated with primary antibodies overnight on the vertical rotor at 4°C. Then, 30 μl of dynabeads® protein A was added to the cell lysate and incubated for another 4 h on the vertical rotor at 4°C. The Dynabeads®-Ab-Ag complexes were washed three times in lysis buffer followed by elution with elution buffer supplied in the kit. Then, the eluted complexes were re-suspended in 4X Laemmli buffer, boiled at 90°C for 10 min and processed further for Western blot analysis as described above.

### Protein-Protein Docking Studies

The protein-protein docking was performed using the ClusPro 2.0 webserver (63, 64). This server performs three computational steps. In the first step, it does rigid-body docking using the PIPER. This docking program is based on the Fast Fourier Transform (FFT) correlation approach and uses pairwise interaction potential as part of its scoring function E = w1E_rep_ + w2E_attr_ + w3E_elec_ + w4EDARS. While E_rep_ and E_attr_ represent the repulsive and attractive contributions, E_elec_ denotes electrostatic energy term and EDARS refers to the pairwise structure-based potential (64, 65). In the second step, 1,000 lowest energy docked structures are clustered using pairwise interface RMSD (IRMSD) (64, 66). Based on the IRMSD values the structure with the highest neighbors within a 9 Å radius is defined as the center of the first cluster. Further clustering is performed within the remaining structures to generate 30 clusters. The energy minimization is done for the structures using the van der Waals terms of the CHARMM potential in the third step (64, 67), following which the structures at the center of the 10 most populated clusters are taken as the output. Since there was no satisfactory template available in PDB to build the homologous model of nsP2, the structure was generated earlier using the I-TASSER algorithm (68). This was used as a ligand in the study. X-ray crystallographic structures of JNK1 (PDB ID: 3ELJ) and p38 (PDB ID: 1A9U) were taken as receptors for the protein-protein docking. These structures were recovered from the protein data bank. The co-crystallized ligands were extracted and energy was minimized before submission of chain A of these structures as receptors. The output of docking generated four types of models using the scoring algorithms designated as balanced, electrostatic-favored, hydrophobic-favored, and van der waals+ electrostatic. Amongst these, the balanced outputs were analyzed. The docking solution with largest members was taken for further visualization using the PyMol software.

### TCR Driven T Cell Activation Assay

Mouse splenocytes isolation and splenic T cell purification from BALB/c mice were performed as reported earlier (69). In brief, using a 70 μM cell strainer the splenocytes were collected from mice spleens. After RBC lysis and washing with 1X PBS, cells were suspended in RPMI-1640 media supplemented with 10% FBS. According to instructions given by the manufacturer's protocol, mouse splenic T cell purification was carried out using Dynabeads Untouched Mouse T Cells Kit (Invitrogen, CA, USA). TCR driven T cell activation was carried out with those purified T cells (CD90.2^+^) in the presence of either CHIKV infected or uninfected (mock) macrophage culture supernatants (0.22 μM membrane filtered) to study the status of CD69 (a T cell activation marker) as described earlier for other infection model (70). For TNF neutralization, anti-TNF purified antibody (BD Bioscience) was incubated for 90 min with the CHIKV infected supernatant prior TCR stimulation.

### Statistical Analysis

Statistical analysis was performed by using the GraphPad Prism 5.0 software (GraphPad Software Inc. USA). Data were represented as Mean ± SEM. The comparison between the groups was performed by either one- or two-way ANOVA with Tukey or Bonferroni *post-hoc* test, respectively. Data presented here were representative of at least three independent experiments. *p* < 0.05 was considered as statistically significant difference between the groups.

## Results

### CHIKV Induces Both p38 and JNK Phosphorylation in Macrophages in a Time-Dependent Manner

**Figure 1:**
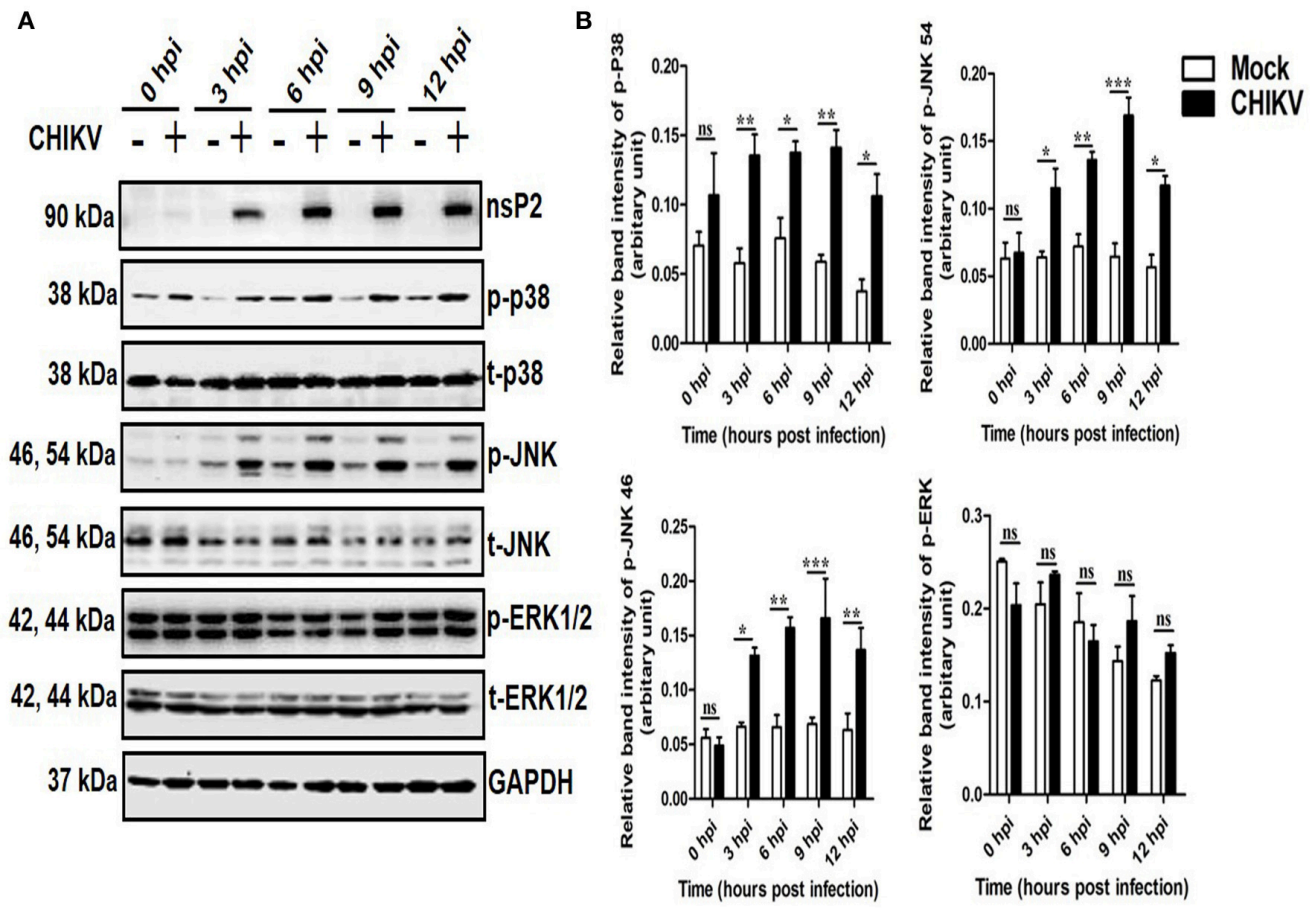
Induction of p-p38 and p-JNK MAPK during CHIKV infection in macrophages. CHIKV infected Raw264.7 cells were harvested at different time intervals followed by Western blot analysis. **(A)** The protein expressions of nsP2, p-p38, t-p38, p-JNK, t-JNK, p-ERK1/2, and t-ERK1/2 were assessed by Western blot analysis. GAPDH was used as loading control. **(B)** Bar diagram showing relative band intensities of p-p38, p-JNK, and p-ERK1/2 at different time post-infection. Data represent mean ± SEM of three independent experiments. *p* < 0.05 was considered as statistically significant difference between the groups. (ns, non-significant; *p < 0.05; **p ≤ 0.01; ***p ≤ 0.001).

To determine whether any MAPK (p38, JNK, and ERK) is activated during CHIKV infection in macrophages, Raw cells were infected with the virus at MOI 5 and harvested at different time points (0–12 hpi). Both the cells and cell culture supernatants were subjected to various downstream assays. As shown in Figure 1A, the p-p38 and p-JNK expressions were increased significantly as compared to the corresponding mock cells. The p-p38 MAPK expression was found to be increased around 1.5-fold as early as 3 and 6 hpi, followed by approximately 3-fold increments toward 12 hpi as compared to the corresponding mock cells. Similarly, the expression of the p-JNK was found to be increased rapidly around 2-fold during early hours (3 and 6 hpi), whereas, it increased up to 3-fold with respect to the mock in later time points. The total p38 and JNK (t-p38 and t-JNK) expressions remain unaffected in both the groups. Moreover, p-ERK1/2 and t-ERK1/2 (total-ERK1/2) expressions remain unchanged throughout all the time points as compared to the corresponding mock (Figures 1A,B). This data suggests that CHIKV induces activation of both p38 and JNK by phosphorylation in a time-dependent manner in macrophages.

### SB203580 Treatment Reduces CHIKV Infection in Macrophages

**Figure 2:**
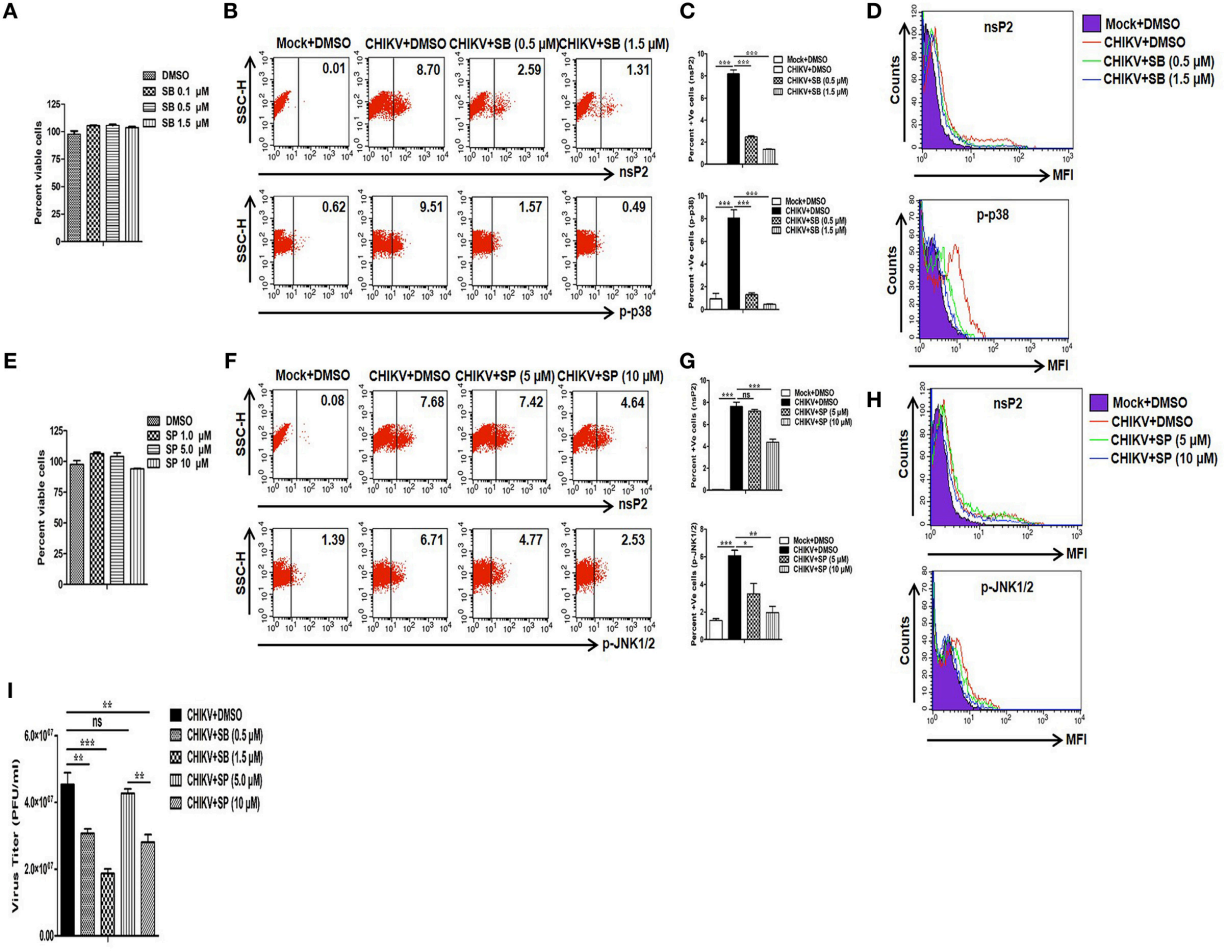
Treatment of SB (p-p38 inhibitor) reduces CHIKV infection in macrophages. CHIKV infected Raw264.7 cells were harvested at 12 hpi with either DMSO or SB or SP treatment followed by flow cytometry and plaque assay based analysis. **(A)** MTT assay showing cytotoxicity of SB in the Raw cell line. **(B)** Dot plot analysis showing expression of nsP2 (upper panel) and p-p38 (lower panel) for mock+DMSO (left), CHIKV+DMSO (middle) and CHIKV+SB (right). **(C)** Bar diagram showing percent positive cells for nsP2 (upper panel) and p-p38 (lower panel). **(D)** MFI of nsP2 (upper panel) and p-p38 (lower panel) for mock+DMSO (purple filled), CHIKV+DMSO (solid red), CHIKV+SB 0.5 μM (solid green) and CHIKV+SB 1.5 μM (solid blue). **(E)** MTT assay showing cytotoxicity of SP in the Raw cell line. **(F)** Dot plot analysis showing expression of nsP2 (upper panel) and p-JNK (lower panel) for mock+DMSO (left), CHIKV+DMSO (middle) and CHIKV+SB (right). **(G)** Bar diagram showing percent positive cells for nsP2 (upper panel) and p-JNK (lower panel). **(H)** MFI of nsP2 (upper panel) and p-JNK (lower panel) for mock+DMSO (purple filled), CHIKV+DMSO (solid red), CHIKV+SP 5.0 μM (solid green) and CHIKV+SP 10 μM (solid blue). **(I)** Bar diagram showing CHIKV titer as PFU/ml in CHIKV+DMSO, CHIKV+SB (0.5 μM and 1.5 μM) and CHIKV+SP (5 μM and 10 μM) at 12 hpi. Data represent mean ± SEM of three independent experiments. *p* < 0.05 was considered as statistically significant difference between the groups. (ns, non-significant; *p < 0.05; **p ≤ 0.01; ***p ≤ 0.001).

Since CHIKV induces both p38 and JNK activation in the host macrophages, next we sought to assess whether these two MAPKs are crucial for the viral infection and replication in the macrophages. For that, pharmaceutical inhibitors of p38 (SB203580) and JNK (SP600125) were used. First, different concentrations of both SB (0.1, 0.5 and 1.5 μM) and SP (1, 5 and 10 μM) were assessed for cytotoxicity in Raw cells by MTT assay. It was observed that around 100% cells were viable in all the concentrations of SB, whereas up to 95 and 100% cells were found to be viable at 10 and 5 μM concentrations of SP, respectively (Figures 2A,E). Thus, both 5 and 10 μM concentrations of SP were selected for further experiments. As SB treatment with >2 μM concentration was known to inhibit phosphorylation and activation of PKB non-specifically (71), both 0.5 and 1.5 μM concentrations of SB was used in the current study.

Raw cells were inoculated with CHIKV in the presence of SB, SP, or solvent control DMSO as described above. At 12 hpi both mock and CHIKV infected cells were harvested and the expressions of nsP2, p-p38, and p-JNK were assessed by Flow cytometry. It was observed that the percent positive cells for nsP2 were reduced from 8.19 ± 0.35 (CHIKV+DMSO) to 2.50 ± 0.08 (CHIKV+SB 0.5 μM) and 1.36 ± 0.02 (CHIKV+SB 1.5 μM), whereas the percent positive cells for p-p38 were reduced from 8.05 ± 0.73 (CHIKV+DMSO) to 1.31 ± 0.15 (CHIKV+SB 0.5 μM) and 0.46 ± 0.04 (CHIKV+SB 1.5 μM) (Figures 2B,C). Likewise, the MFI for both the p-p38 and nsP2 were reduced at 12 hpi in the SB treated cells as compared to the DMSO control (Figure 2D). The inhibition of p-JNK by SP at 5.0 μM concentration did not affect nsP2 expression in the macrophages as compared to the DMSO control (CHIKV+DMSO; 7.63 ± 0.40, CHIKV+SP 5 μM; 7.21 ± 0.17, *p* > 0.05), despite significant reduction in the p-JNK percent positive cells (CHIKV+DMSO; 6.08 ± 0.40, CHIKV+SP; 3.31 ± 0.75, *p* < 0.05). However, SP at the comparatively higher concentration (10 μM) did reduces nsP2 expression by around 1.5-fold (Figures 2F–H). Further, plaque assay of the cell culture supernatants revealed that SB treatment reduces the number of new viral progeny release around 1.5- and 2.5-fold at 0.5 and 1.5 μM, respectively. Whereas, SP at 10 μM concentration treatment reduces the number of new viral progeny release around 1.6-fold as compared to the corresponding DMSO control (Figure 2I). This result indicates that the activation of both p38 and JNK MAPKs might be crucial for the CHIKV infection and replication in the host macrophages with SB being more effective comparatively in controlling infection than SP.

### Pharmaceutical Inhibitors Specific to p-p38 and p-JNK Reduces CHIKV Induced TNF Production in the Host Macrophages

**Figure 3:**
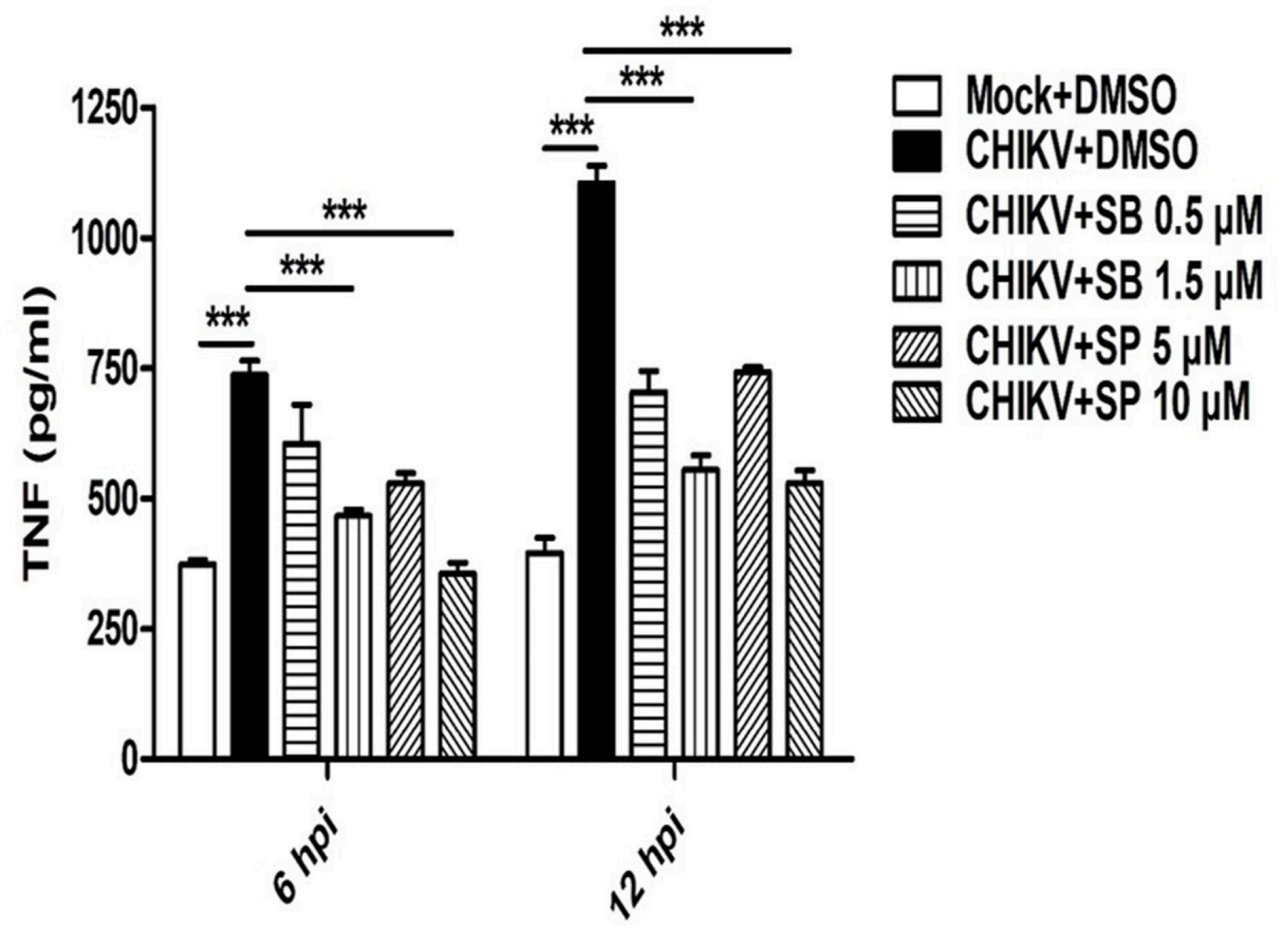
SB and SP both reduce CHIKV induced TNF in the host macrophages. Raw264.7 cells were infected with CHIKV at MOI 5. The cells were treated with either DMSO or SB or SP at different concentrations as described earlier. Bar diagram depicting production of TNF (pg/ml) at 6 and 12 hpi with mock+DMSO, CHIKV+DMSO, CHIKV+SB (0.5 μM and 1.5 μM) and CHIKV+SP (5 μM and 10 μM). Data represent mean±SEM of three independent experiments. *p* < 0.05 was considered as statistically significant difference between the groups. (***p ≤ 0.001).

Activation of MAPKs by different pathogens has been shown to induce pro-inflammatory cytokines such as TNF in the host cells (50, 54). Since, CHIKV triggers robust TNF production (a key mediator of inflammation) in the host macrophages (31, 37), it was interesting to investigate whether any MAPKs are involved in this pathway. Accordingly, macrophages were treated with either SB or SP and infected with CHIKV as mentioned earlier. The cell culture supernatants were subjected to sandwich ELISA for the detection of TNF at early (6 hpi) and late (12 hpi) time post-infection. It was observed that both SB and SP could suppress CHIKV induced TNF significantly at both the time points as compared to the corresponding DMSO control. At 6 hpi the TNF level for CHIKV+DMSO was found to be 737 ± 27 pg/ml (mean ± SEM), which was reduced to 466 ± 12 pg/ml (mean ± SEM, *p* < 0.05) and 356 ± 20 pg/ml (mean ± SEM, *p* < 0.05) in the presence of SB (1.5 μM) and SP (10.0 μM), respectively. Similarly, at 12 hpi, the TNF production was 1,104 ± 29 pg/ml (mean ± SEM) in the CHIKV+DMSO sample, whereas it was reduced to 554 ± 28 pg/ml (mean ± SEM, *p* < 0.05) for SB and 528 ± 25 pg/ml (mean ± SEM, *p* < 0.05) for SP treatment (Figure 3). Taken together, this result suggests that CHIKV might induce TNF via p38 as well as JNK mediated pathways in the host macrophages.

### CHIKV Induced TNF Facilitates TCR Driven T Cell Activation

**Figure 4:**
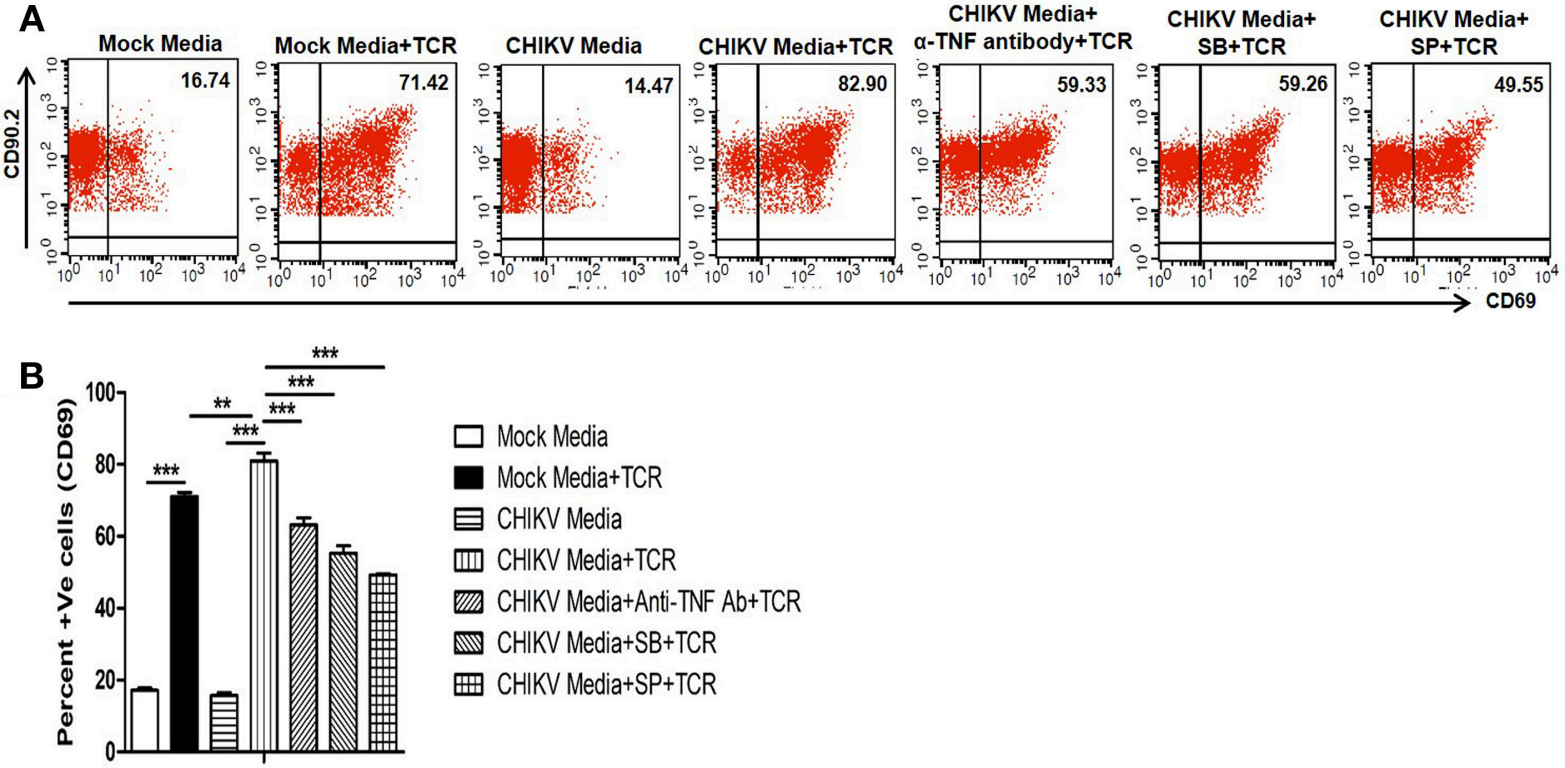
CHIKV induced TNF facilitates TCR driven T cell activation *in vitro*. Both CHIKV infected and mock cell culture supernatants were harvested and used to culture for T cells activation assay *in vitro*. **(A)** Dot plot analysis showing the expression of CD69 in different conditions. **(B)** Graphical representation depicting the percent positive cells for CD69 in T cells. Data represent mean ± SEM of three independent experiments. *p* < 0.05 was considered as statistically significant difference between the groups. (**p ≤ 0.01; ***p ≤ 0.001).

TNF, one of the potent inflammatory cytokine, which can enhance TCR-dependent T cell activation (72). We and others have previously reported that *in vitro* CHIKV infection in RAW 264.7 cells leads to TNF production (31, 37). Recent studies have shown a pathogenic role of T cells during CHIKV infection associated to host inflammatory responses (43, 44, 46). Here we have investigated whether CHIKV infection induced macrophage derived TNF can facilitate mouse T cell activation associated with cell mediated immunity. For this, CHIKV infected culture supernatant of RAW 264.7 cells were tested toward TCR driven resting T cell activation assay (69). We have found that CHIKV infected macrophage culture supernatant along with TCR activation facilitated the induction of CD69 level (around 81%) as compared to uninfected culture supernatant (around 71%). Interestingly, when the CHIKV infected macrophage culture supernatant was treated with TNF neutralizing antibody, a sharp decrease of CD69 frequency (around 63%) was observed. Beside this, SB and SP treated CHIKV infected Raw 264.7 culture supernatant along with TCR stimulation also showed downregulation of CD69 frequency in T cells (Figures 4A,B). So, the above observations may underscore that the TNF present in CHIKV infected culture supernatant might be able to facilitate the induction of T cell activation.

### CHIKV Infection Induces Key Transcription Factors in the Host Macrophages

**Figure 5:**
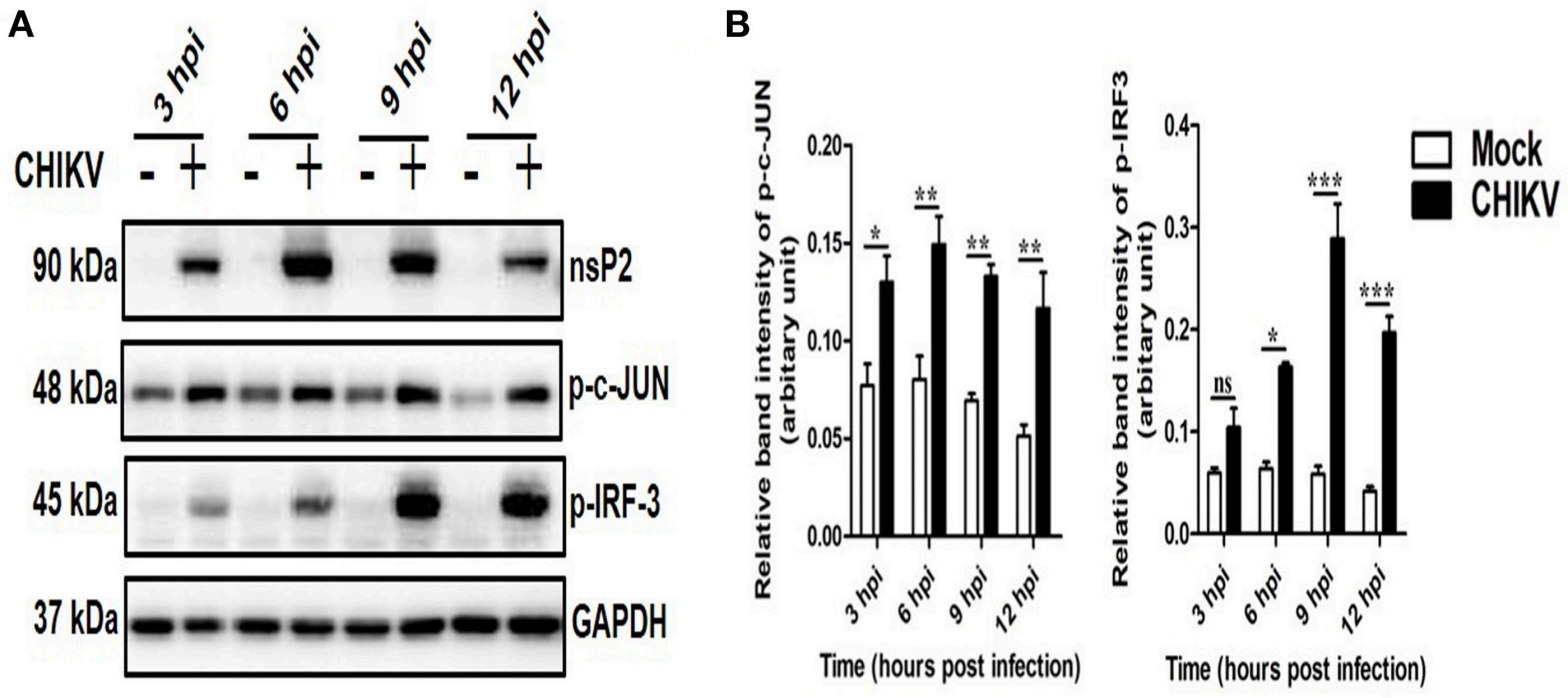
Induction of key transcription factors by CHIKV in macrophages. CHIKV infected Raw264.7 cells were harvested at different time intervals followed by Western blot analysis. **(A)** Western blot analysis depicting p-c-jun and p-IRF-3 protein expressions at different time post-infection. GAPDH serves as loading control. **(B)** Bar diagram showing relative band intensities of p-c-jun and p-IRF3 at different times post-infection. Data represent mean ± SEM of three independent experiments. *p* < 0.05 was considered as statistically significant difference between the groups. (ns, non-significant; *p < 0.05; **p ≤ 0.01; ***p ≤ 0.001).

Often, the viral infection is associated with the activation and localization of several transcription factors (e.g., IRFs, c-jun, p53), which in turn regulates host responses to viruses (73–78). Here, the expressions of key transcription factors involved mainly in antiviral responses (p-IRF3) and TNF production (p-c-jun) were assessed at different hpi by Western blot analysis. It was observed that both p-IRF3 and p-c-jun were induced significantly in the CHIKV infected macrophages as compared to the corresponding mock (Figures 5A,B). This data suggest that CHIKV infection in the Raw cell line might be associated with the elevation of key antiviral and inflammatory transcription factors.

### CHIKV Induces p-c-Jun via JNK MAPK Activation in Macrophages

**Figure 6:**
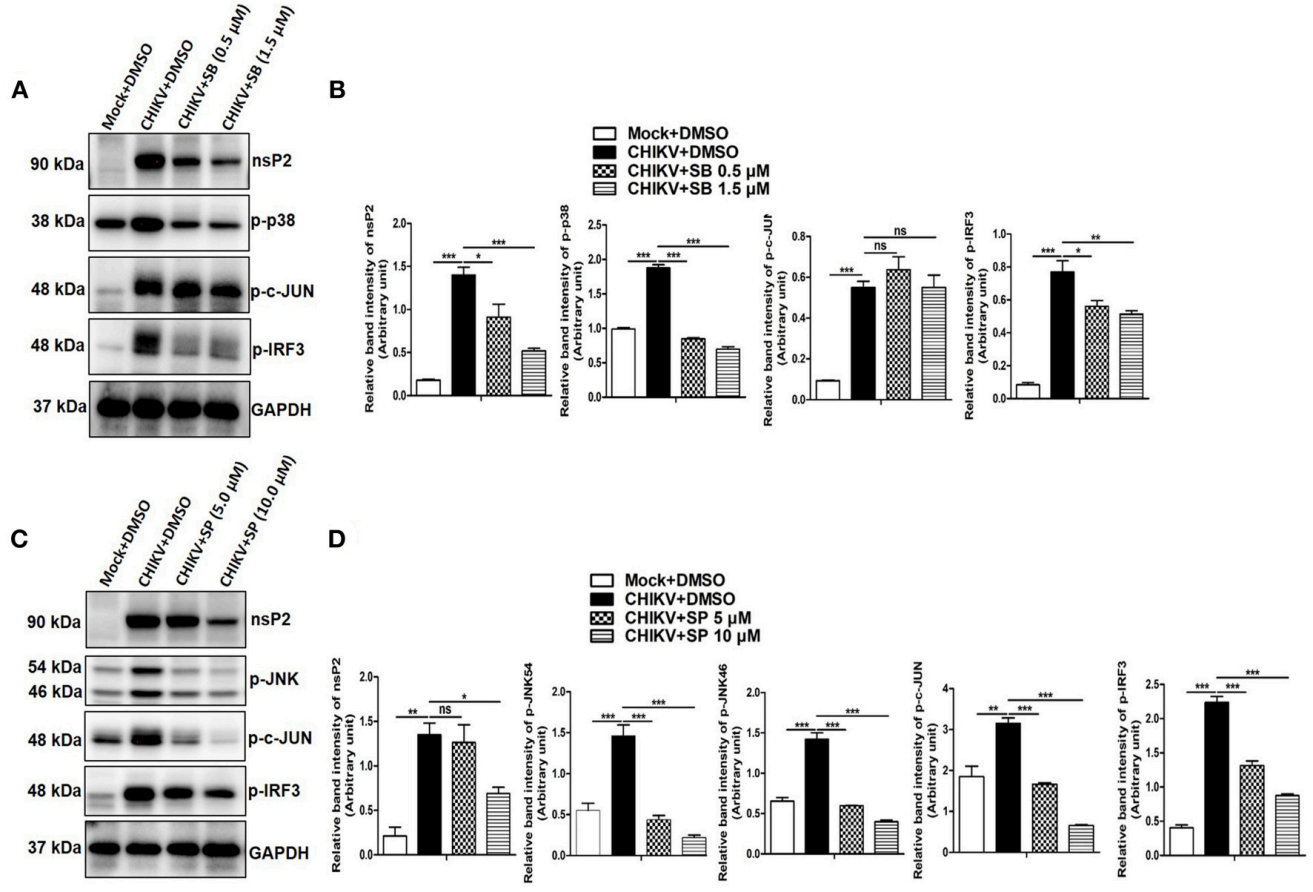
CHIKV induces p-c-Jun via JNK MAPK activation in macrophages. Raw264.7 cells were infected with CHIKV at MOI 5. The cells were treated with either DMSO or SB (0.5 and 1.5 μM) or SP (5.0 and 10 μM) as described earlier. Both mock and CHIKV infected Raw264.7 cells were harvested at 12 hpi followed by Western blot analysis. **(A)** Western blot analysis depicting nsP2, p-p38, p-c-jun and p-IRF3 protein expressions for mock+DMSO, CHIKV+DMSO, and CHIKV+SB. **(B)** Bar diagram showing relative band intensities of nsP2, p-p38, p-c-jun and p-IRF3 for mock+DMSO, CHIKV+DMSO, and CHIKV+SB at 12 hpi. **(C)** Western blot analysis depicting nsP2, p-JNK, p-c-jun, and p-IRF3 proteins expressions for mock+DMSO, CHIKV+DMSO and CHIKV+SP. **(D)** Bar diagram showing relative band intensities of nsP2, p-JNK, p-c-jun, and p-IRF3 for mock+DMSO, CHIKV+DMSO, and CHIKV+SP at 12 hpi. GAPDH serves as loading control. Data represent mean ± SEM of three independent experiments. *p* < 0.05 was considered as statistically significant difference between the groups. (ns, non-significant; *p < 0.05; **p ≤ 0.01; ***p ≤ 0.001).

It has been reported previously that TNF is one of the key mediators for arthritis or arthritis-like diseases in humans by promoting severe inflammation. Although, several other inflammatory cytokines are elevated in RA (rheumatoid arthritis), anti-TNF therapy seems to be promising for the effective treatment against it (79). Since CHIKV induces TNF via p38/JNK MAP kinase pathways and phosphorylation of c-jun is reported to be associated with TNF production in other inflammatory model system (50, 80), phosphorylation of c-jun in both mock and CHIKV infected macrophages was assessed by Western blot analysis. Surprisingly, the expression of p-c-jun was reduced around 1.8- and 4.77-fold in the presence of SP at 5 and 10 μM indicating a plausible role of JNK toward c-jun phosphorylation, whereas SB treatment at 1.5 μM did not affect p-c-jun expression significantly. However, both SB and SP treatment suppressed p-IRF-3 expression which is induced by CHIKV as compared to the DMSO control (Figures 6A–D). Taken together, the current data depict that CHIKV may induce p-c-jun via JNK pathway whereas induction of p-IRF-3 might be dependent on both p38 and JNK MAPKs.

### CHIKV nsP2 Interacts With Host p-p38 and p-JNK MAPKs in the Macrophages

**Figure 7:**
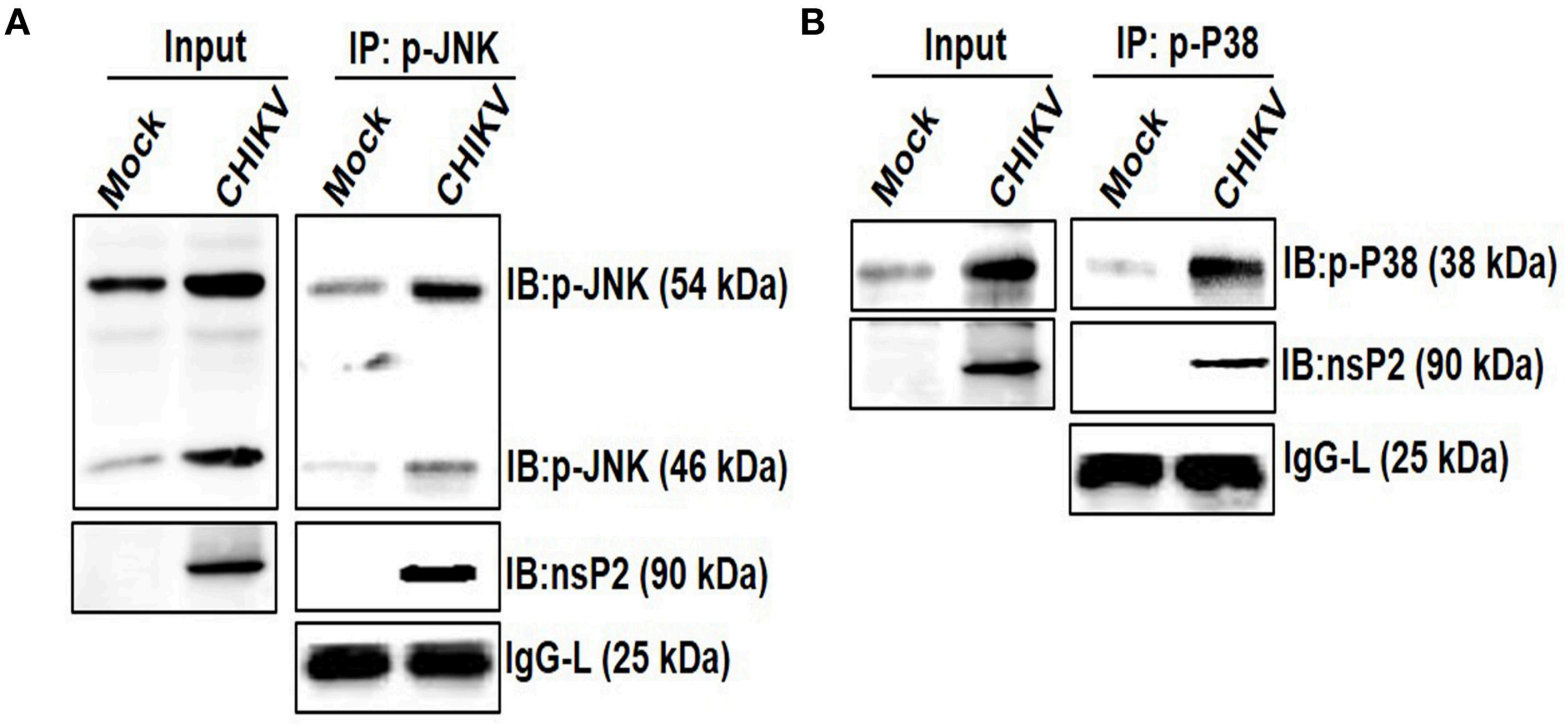
Interaction of CHIKV nsP2 with p-p38 and p-JNK MAPKs in the host macrophages. Raw264.7 cells were infected with CHIKV at MOI 5. Both mock and CHIKV infected Raw264.7 cells were harvested at 6 hpi and processed for IP as per the protocol mentioned in the materials and methods followed by Western blot analysis. **(A)** Western blot analysis depicting the expressions of nsP2 and p-JNK in the whole cell lysate (left), co-immunoprecipitation analysis showing the interaction of CHIKV nsP2 and p-JNK in the host macrophages (right) **(B)** Western blot analysis depicting the expression of nsP2 and p-p38 in the whole cell lysate (left), co-immunoprecipitation analysis showing the interaction of CHIKV nsP2 and p-p38 in the host macrophages (right).

Viruses are small obligatory intracellular pathogens utilizes the metabolic pathways of the host for replication. Very often viruses also shut-off host translational process, which might be a strategic decision to contain antiviral responses (81, 82). The integration of complex proteomics studies including *in silico* protein-protein interaction predictions keeps on unraveling the complex network of interaction with the host cell proteins. Throughout the course of replication, these pathways rely heavily on the dynamic and temporarily regulated virus-host protein-protein interactions which are crucial for the virus replication, pathogenesis, and viral subversion of host defense. The identification and characterization of these interacting partners also help in the delineation of the viral protein functions precisely and might be very helpful in designing rationale drugs for an effective treatment (83–85). The interaction of host MAPK with viral protein has been shown earlier, which in turn regulates infection and replications (86). Since CHIKV infection modulated the phosphorylation of host p38 and JNK, their interaction with the nsP2 protein was investigated. For that, Raw cells were infected with CHIKV and harvested at 6 hpi for further analysis. Co-immunoprecipitation followed by Western blot analysis showed that both the p-p38 and p-JNK proteins were pulled with the CHIKV-nsP2 protein in the host macrophages (Figures 7A,B). This result indicates that CHIKV-nsP2 interacts with both p-p38 and p-JNK and this might be playing a crucial role in the CHIKV infection and TNF mediated inflammatory responses.

### Protein-Protein Docking Analysis Shows the Specific Amino Acids Responsible for the nsP2-MAPK Interactions

**Figure 8:**
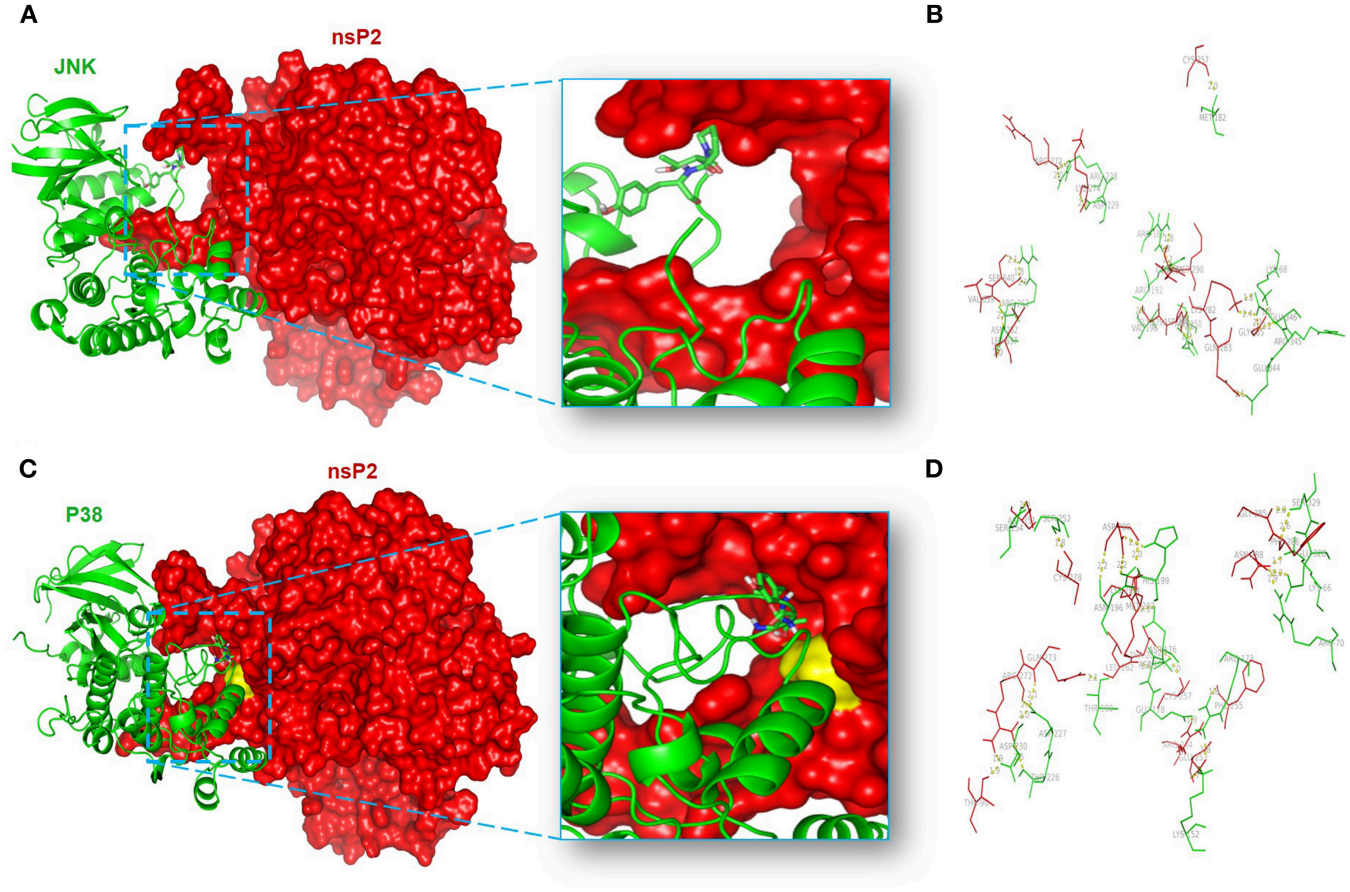
Protein-protein docking analysis shows the probable interaction of CHIKV-nsP2 with host p38 and JNK1. The protein-protein docking was performed using the ClusPro 2.0 web server. **(A)** Model showing the probable interaction of nsP2 (red surface) with host JNK1 (green ribbon). **(B)** Figure highlights polar interactions (yellow bridge) between residues of nsP2 (red) and host JNK1 (green). **(C)** Model showing probable interaction of nsP2 (red surface) with host p38 (green ribbon). The phosphorylation lip of p38 (element colored stick residues) shows close proximity to Gln-273 (yellow surface) of nsP2. **(D)** Figure depicts polar interactions (yellow bridge) between residues of nsP2 (red) and p38 (green).

In order to unravel the amino acid residues responsible for the interaction of nsP2-MAPKs, protein-protein docking was carried out as mentioned above (64, 87, 88). The balanced outputs were preferred from the docking results as this mode takes into account all possible modes of interactions. The most stable complex of nsP2-JNK1 on visualization by using the PyMol software suggested the possible involvement of different residues in the interaction (Supplementary Table S1). No interaction was found between the phosphorylation lip (Thr-183-X-Tyr-185) of JNK1 and nsP2 (Figure 8A). This suggests a poor fit of JNK1 active site with nsP2. Nonetheless, ten polar interactions were observed within 2å (Figure 8B). Some of these include the interactions of Met-182, Arg-228, Arg-189, Val-196, Arg-150, Lys-68, and Glu-346 of JNK1 with Cys-217, Arg-272, Gln-291, Gly-279, Asp-280, Gly-285, and Lys-282 of nsP2, respectively (Figure 8B). The most stable complex of nsP2-p38 showed a close fit of the phosphorylation lip (Thr-180-X-Tyr-182). In addition to that, a polar interaction was suggested between Thr-180 of p38 and Gln-273 of nsP2 (Figure 8C). Some of the polar interactions were also observed between Lys-66, Ser-329, Asn-196, Ser-252, Ser-254, Asp-177, Glu-178, Arg-173, Lys-152, and Asp-230 of p38 and Asn-288, Gly-285, Asp-280, Cys-278, Asp-351, Cys-257, Arg-244, Phe-255, Arg-272, and Thr-90 of nsP2, respectively (Figure 8D and Supplementary Table S2). Thus, these results further suggest that CHIKV-nsP2 interacts with p38 as well as JNK MAPKs during viral infection in the host macrophages. Moreover, the phosphorylation lip of p38 interacts more closely with the Gln-273 of CHIKV-nsP2, which supports the findings of IP experiments.

## Discussion

The recent epidemics of Chikungunya virus (CHIKV) with unprecedented magnitude and unusual clinical severity have raised a great public health concern worldwide, due to the absence of a vaccine or specific anti-CHIKV therapy. TNF is one of the robustly induced cytokine by CHIKV and in the current study, we have investigated the molecular mechanism involved in the induction of TNF in the host macrophages. Our data suggested that CHIKV induces both p38 and JNK phosphorylation in macrophages in a time-dependent manner. Moreover, p-p38 and p-JNK inhibition by SB and SP were found to reduce CHIKV infection. Interestingly, SB mediated inhibition of CHIKV infection was found to be more effective even at lower concentration as compared to SP. Further, inhibition of both p-p38 and p-JNK reduced CHIKV induced TNF in the host macrophages. Moreover, CHIKV infected cell culture supernatant is found to facilitates T cell activation via TNF in TCR primed T cells. Besides, it was observed that the expressions of key transcription factors involved mainly in antiviral responses (p-IRF3) and TNF production (p-c-jun) were induced significantly in the CHIKV infected macrophages as compared to the corresponding mock cells. Further, it was found that CHIKV mediated TNF production in the macrophages is dependent on p38 and JNK MAPK pathways linking p-c-jun transcription factor. Interestingly, it was also noticed that CHIKV nsP2 interacts with host p-p38 and p-JNK MAPKs in the macrophages. This observation was supported by the *in silico* protein-protein docking analysis which illustrates the specific amino acids responsible for the nsP2-MAPKs interactions and a strong polar interaction was predicted between Thr-180 (within the phosphorylation lip) of p38 and Gln-273 of nsP2. However, no such polar interaction was predicted for the phosphorylation lip of JNK which indicates the differential roles of p-p38 and p-JNK during CHIKV infection in the host macrophages.

The MAPKs have been shown to be activated by several viral infections (54–57). Using the mouse macrophage cell line, Raw264.7 cells, we report for the first time that CHIKV induces both p-p38 and p-JNK significantly, however, the p-ERK1/2 expression remains unchanged. Interestingly, the up-regulation of p-ERK has been reported earlier during CHIKV infection in non-immune BHK cell lines (60). Another report suggested that, the nuclear localization of ERK1/2 (un-phosphorylated form) in the uninfected microglia cells increases after CHIKV infection in astrocytes and this might be due to the release of some factor(s) from infected astrocytes *in vitro* (59).

In this study, it was found that inhibition of p38 signaling by SB reduces nsP2 protein expression and new viral progeny release remarkably, whereas inhibition of JNK signaling by higher concentration of SP could reduce nsP2 moderately as compared to DMSO control. This result indicates that both p38 and JNK play pro-viral role in CHIKV infection in the host macrophages and similar observations have been reported previously in case of other viral infections (60, 89–91). The Encephalomyocarditis Virus infection was suppressed in L929 cells by SB, mainly through the reduction of the viral protein synthesis (89). Whereas, in the Human Enterovirus 71 infection it was shown that the blockage of virus induced p-p38 leads to significant reduction in both viral protein and progeny release (90). Further investigation can be carried out on other CHIKV proteins and RNA synthesis to understand the pro-viral role of the p-p38 in viral replication in details.

**Figure 9:**
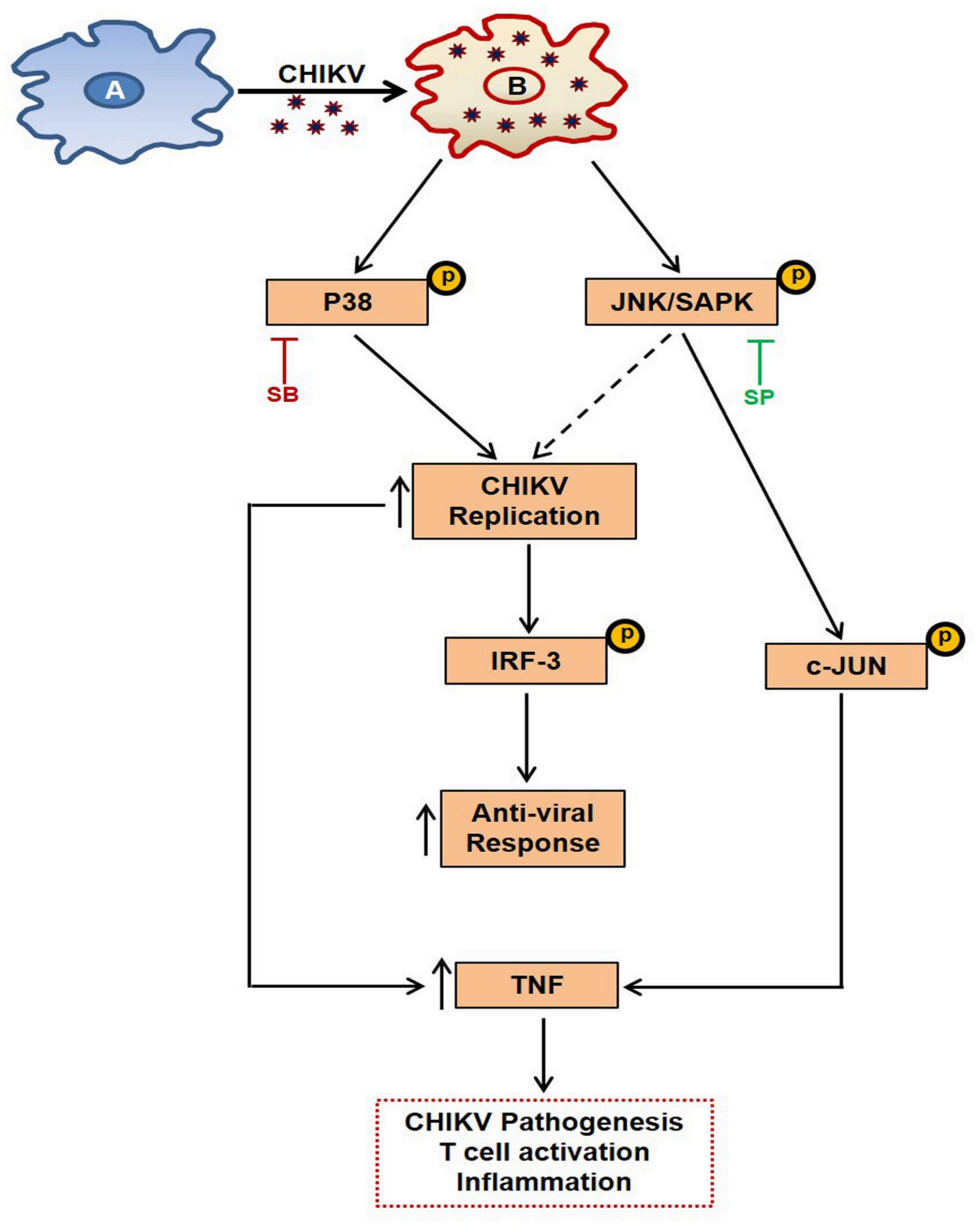
Schematic representation of the proposed working model showing the involvement of MAPK pathways during CHIKV infection in macrophages. **(A)** Uninfected macrophage. **(B)** CHIKV infected macrophage depicting activation of p-p38 and p-JNK, leading to induction of TNF and anti-viral response which has been elaborated in the text.

MAPKs are known to regulate TNF production via p-c-jun in other inflammation models (50). Here, it was observed that the expression of p-c-jun is dependent on p-JNK pathway (as SP reduces p-c-jun expression in a dose dependent manner), whereas induction of p-IRF3 is dependent on both MAPKs (p38 and JNK) during CHIKV infection in macrophages. Therefore, it is quite possible that the p-JNK pathway induction by CHIKV leads to the activation of antiviral responses via p-IRF3 and pro-inflammatory responses (TNF) via p-c-jun pathway. On the other hand, p-p38 is involved in activating both pro-viral and anti-viral pathways (via induction of p-IRF3) (Figure 9). It has also been observed during this investigation that pro-inflammatory TNF production was decreased significantly during SB treatment. This might be due to the marked inhibition of CHIKV infection, however the possibilities of the involvement of other factors cannot be ignored.

TNF may promote the activation and proliferation of T cells and thereby regulate the overall T cell mediated effector function (72). In mouse model system, it has been demonstrated that host T cells are induced during experimental CHIKV infection and are associated with CHIKV mediated pathogenesis (43, 44, 46). In the present study, we found that CHIKV infected macrophage culture supernatant may facilitate TCR driven activation of resting T cells as compared to the mock supernatant. Further, the use of neutralizing anti-TNF antibody towards the regulation of the T cell activation suggests that it could be mediated via CHIKV induced macrophage derived TNF. Additionally, presence of either SB or SP in the CHIKV infected macrophage supernatant also able to reduce T cell activation *in vitro*, indicating an effect of macrophage derived TNF on T cell activation during CHIKV infection. Except few cases, CHIKV is not fatal, however, the long-term polyarthralgia, arthritis-like symptoms along with severe inflammation remain a concern for most of the chronic patients (20, 25, 92–95). TNF is one of the key mediator of arthritis or arthritis-like diseases in humans by triggering severe inflammation. Despite the elevation of several other inflammatory cytokines in RA, anti-TNF therapy holds a promise for the effective treatment against it (96, 97), which might be exploited against CHIKV pathogenesis in future.

Further, the co-immunoprecipitation analysis revealed that CHIKV-nsP2 interacts with both p-p38 and p-JNK upon infection in the host macrophages. This was also supported by the *in silico* analysis of the protein-protein interaction of CHIKV-nsP2 with p38 and JNK. The phosphorylation lip of p38 was found to interact with nsP2 due to the observed close fit model and a polar interaction between Thr-180 of p38 and Gln-273 of nsP2. Residues from the N-terminus of nsP2 were also suggested to have strong (<2 Å) polar interactions with the other residues around this active site. Unlike this, the interaction of CHIKV-nsP2 showed poor fit with the phosphorylation lip of JNK and close (<2 Å) polar interaction was also observed for residues from N-terminus of nsP2. These interactions might be one of the yet unknown strategies to utilize host signaling pathways through protein-protein interactions for effective viral infection (97–99), which can be explored further in details.

Viral proteins are found to be phosphorylated by various kinases, which in turn regulate its functions, stability and interactions with other cellular and viral proteins (100). However, the precise role of the viral protein phosphorylation (especially in Alphavirus) has not been reported yet. In this investigation, nsP2 was found to interact with the phosphorylation lip of p38, hence, *in silico* analysis was carried out using PTM prediction tools, GPS (group-based phosphorylation scoring method) (101, 102) and NetPhos 3.1, to predict the target phosphorylation sites of nsP2 in a kinase specific manner (103). The GPS is a group-based phosphorylation algorithm, which predicts kinase-specific phosphorylation sites among different host protein kinase groups according to specific sequence pattern (101, 102). Whereas, the NetPhos server is based on an artificial neuronal network (ANN) that allows the users to choose between generic predictions based on the given protein sequence or kinase-specific predictions (103, 104). Out of several predictions, both the softwares predicted T5, S28, and S513 sites in CHIKV-nsP2 with a high probability of phosphorylation by p38 (Supplementary Table S3). Further, to elucidate whether positions of these amino acids in CHIKV-nsP2 is associated with any consensus regions of functional importance, the predicted peptides were searched in the ExPASY-PROSITE protein database. Surprisingly, the peptide “FKEDKAYSPEVALNE” with S513 (at the middle, red) showed a hit with Alphavirus nsP2 protease domain belonging to the C9 cysteine protease family (105). Since we have shown earlier that p38 interacts strongly with CHIKV nsP2 (with phosphorylation lip) and the inhibition of p38 activation strongly reduces CHIKV infection, it might be possible that, p38 phosphorylates either nsP2 directly or through the association of other client protein(s) which in turn may modulate its function. However, further studies are required to corroborate the CHIKV-nsP2 phosphorylation by host kinases and its functional consequences on infection and pathogenesis.

In summary, for the first time it has been shown that CHIKV triggers robust TNF production (a key mediator of CHIKV induced inflammation) in the host macrophages via both p-p38 and p-JNK/p-c-jun pathways and viral protein nsP2 interacts with both the MAPKs during infection. Furthermore, CHIKV induced macrophage derived TNF was found to facilitate T cell activation *in vitro*. Hence, this information might shed light in rationale-based drug designing for the control of the disease caused by CHIKV in future.

## Author Contributions

SuC, SoC, TN, and PM conceived the idea and designed the experiments. TN, PM, SS, PK, SC, and CM performed wet lab experiments. BS, TN, and PM performed *in silico* experiments and analysis. SuC and SoC contributed reagents. SuC, SoC, TN, and PM analyzed and interpreted the data. SuC, SoC, TN, PM, CM, SC, and BS wrote the manuscript. All authors read and approved the final version of this manuscript.

### Conflict of Interest Statement

The authors declare that the research was conducted in the absence of any commercial or financial relationships that could be construed as a potential conflict of interest.
