# Supplementary material for: P38 and JNK Mitogen-Activated Protein Kinases Interact With Chikungunya Virus Non-structural Protein-2 and Regulate TNF Induction During Viral Infection in Macrophages
Source: Front Immunol. 2019 Apr 12;10:786. doi: 10.3389/fimmu.2019.00786 (PMC6473476; doi:10.3389/fimmu.2019.00786)
Supplement: Supplementary file 1 [file Data_Sheet_1.pdf]

Supplementary Material

P38 and JNK Mitogen-Activated Protein Kinases Interact with Chikungunya Virus Non-Structural Protein-2 and Regulate TNF Induction during Viral Infection in Macrophages

Tapas Kumar Nayak, Prabhudutta Mamidi, Subhransu Sekhar Sahoo, P. Sanjai Kumar, Chandan Mahish, Sanchari Chatterjee, Bharat Bhusan Subudhi, Soma Chattopadhyay, Subhasis Chattopadhyay*

*** Correspondence:** Dr. Subhasis Chattopadhyay: subho@niser.ac.in

Supplementary Tables

Supplementary Table S1. Different polar interactions between residues of JNK1 and nsP2 with H-bond distances in Å.

JNK1 (PDB ID: 3ELJ)	H- bond (Å)	NsP2
Met-182	2.0	Cys-217
Asp-229	2.5	Lys-274
Arg-228	2.0	Arg-272
Arg-228	2.4	Arg-272
Asn-262	2.0	Leu-637
Asn-262	2.4	Leu-637
Asn-262	2.4	Val-639
Arg-263	2.1	Ser-640
Arg-263	1.8	Gln-291
Arg-189	1.8	Gln-291
Arg-189	2.2	Met-290
Val-196	2.0	Gly-279
Arg-150	1.9	Asp-280
Arg-150	1.9	Asp-280
Glu-344	2.1	Gln-283
Arg-345	2.2	Gly-285
Lys-68	1.8	Gly-285
Glu-346	2.1	Gly-285
Glu-346	2.4	Lys-282
Glu-346	1.8	Lys-282

Supplementary Table S2. Different polar interactions between residues of P38 and nsP2 with H-bond distances in Å.

P38 (PDB ID: 5NZZ(E))	H bond (Å)	nsP2
Lys-15	1.7	Tyr-544
Lys-15	1.8	Asp-546
Gln-60	1.9	Asn-547
Ser-261	1.9	Tyr-666
His-228	3.2	His-779
Ser-254	2.6	Arg-736
Glu-178	1.9	Arg-749
Asp-331	2.1	Asn-771
Asp-331	1.9	Arg-774
Asp-331	2.1	Arg-774
Tyr-182	2.4	Asp-550
Tyr-182	2.0	Asn-551
Arg-186	1.8	Asp-550

Supplementary Table S3. Phosphorylation sites predicted by both the GPS software and the NetPhos server for P38 MAP kinase of nsP2 protein of Chikungunya virus.

Si no.	GPS (group based phosphorylation scoring method) based predictions		
	**Position**	**Peptide**	**Amino acid**
1	5	***GIIETPRGAIKV	T
2	28	VGEYLVLSPQTVLRS	S
3	513	FKEDKAYSPEVALNE	S
**NetPhos based predictions**			
	**Position**	**Peptide**	**Amino acid**
1	5	GIIETPRGA	T
2	28	YLVLSPQTV	S
3	513	DKAYSPEVA	S
